# Band Alignment and Interfacial Stability of Co_3_O_4_ vs NiO as a Hole Transport Layer with FA_0.4_MA_0.6_PbI_3_ Perovskite

**DOI:** 10.1021/acsami.4c20008

**Published:** 2025-04-08

**Authors:** Xuewei Zhang, Xiaxia Cui, Qidong Tai, Daping Chu, Yuzheng Guo, John Robertson

**Affiliations:** † Laboratory for Computational Engineering, Swiss Federal Laboratories for Materials Science and Technology, Empa institution, Dübendorf 8600, Switzerland; ‡ Department of Engineering, University of Cambridge, Cambridge CB2 1PZ, United Kingdom; § The Institute of Technological Sciences, Wuhan University, Wuhan 430072, China; ∥ Hubei Key Laboratory of Electronic Manufacturing and Packaging Integration, Wuhan University, Wuhan 430072, China; ⊥ School of artificial intelligence, Anhui University of Science and Technology, Huainan, Anhui 232000, China

**Keywords:** halide perovskite, hole transport layer, interface, band alignment, stability, density functional theory, photovoltaic

## Abstract

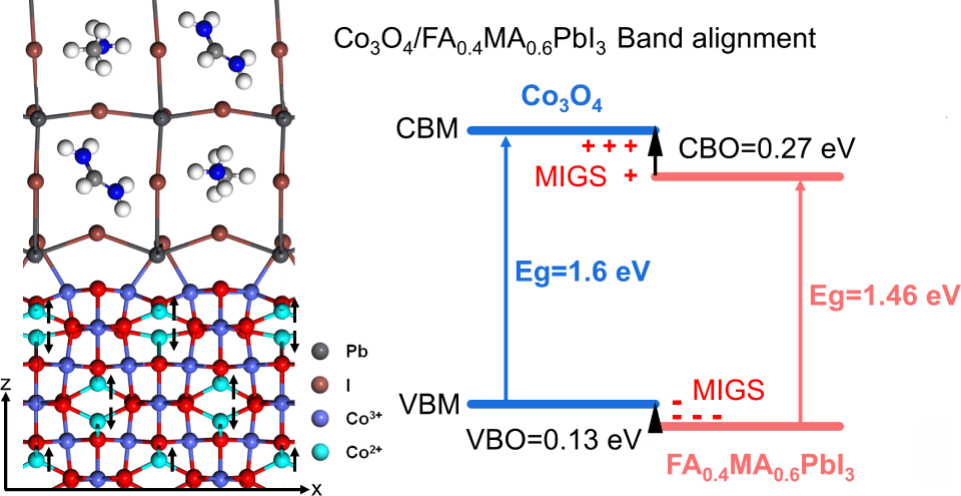

The unstable cubic phase of halide perovskites (ABX_3_) and the poor interfacial quality between their absorbing layer and the hole transport layer (HTL) cause the long-term instability of halide perovskite solar cells (PSCs). To stabilize the intrinsic cubic perovskite structure, mixing CH_3_NH^+^ (MA^+^) and CH(NH_2_)^+^ (FA^+^) large organic ions at the A site is frequently used. Although NiO offers better stability than organic HTLs, such as poly(triaryl-amine) (PTAA), the stability of NiO-based PSCs still remains an issue, primarily due to the formation of interfacial Ni vacancies at the NiO/perovskite interface. In this theoretical study, by analyzing Co_3_O_4_/FA_0.4_MA_0.6_PbI_3_ and NiO/perovskite interfaces, we show that Co_3_O_4_ offers greater benefits as an HTL material than NiO for three main reasons. First, Co_3_O_4_/FA_0.4_MA_0.6_PbI_3_ shows a type II band alignment with a small valence band offset (0.13 eV), whereas NiO/FA_0.4_MA_0.6_PbI_3_ interfaces give type I band alignments. Second, Co_3_O_4_/FA_0.4_MA_0.6_PbI_3_ interfaces show higher adhesion energy (1.48 J/m^2^) than NiO/FA_0.4_MA_0.6_PbI_3_ interfaces, indicating enhanced interfacial stability. Third, the formation of interfacial Co vacancies in NiO/FA_0.4_MA_0.6_PbI_3_ presents greater difficulty due to their higher formation energy of 1.75 eV compared to the Ni vacancies in NiO/FA_0.4_MA_0.6_PbI_3_, suggesting better stability under environmental conditions. FA_0.4_MA_0.6_PbI_3_ also shows higher adhesion energies with Co_3_O_4_ or NiO than those for MAPbI_3_. Therefore, we suggest that the combination of Co_3_O_4_ as the HTL and FA_0.4_MA_0.6_PbI_3_ as the light-absorbing layer holds great potential for achieving PSCs with long-term stability.

## 1 Introduction

Halide perovskites belong to a class of materials having the chemical formula ABX_3_, where the A-site cation can be Cs, CH(NH_2_)_2_^+^ (FA^+^), or CH_3_NH_3_^+^ (MA^+^), the B-site cation can be Pb^2+^ or Sn^2+^, and the X-site anion can be I^–^ or Br^–^ or Cl^–^. Halide perovskites are one of the most promising next-generation photovoltaic materials for solar cells as a replacement of silicon, due to their thin-film fabrication, easy synthesis, low-cost production, high light-absorption ability, and high carrier mobility.^1−7^ Over the past 15 years, the power conversion efficiency (PCE) of these solar cells has surged from 3.8% in 2009^2^ to an impressive 30% in 2023,^8^ including tandem architectures with silicon. However, the instability of halide perovskite solar cells (PSCs), especially under environmental conditions, causes severe degradation, hindering their long-term usage.^1^

Widely applied PSCs in the p-i-n structure consist of five key components: an anode, a hole transport layer (HTL), a light-absorbing layer (halide perovskites), an electron transport layer (ETL), and a cathode.^1,9^ When the solar cell is exposed to sunlight, photons excite electrons from the valence band (VB) to the conduction band (CB). Separated electrons and holes migrate from within the perovskite layer toward its interfaces with the ETL and HTL,^1^ respectively. Subsequently, electrons and holes are transported by the ETL and HTL and collected by the cathode and anode, respectively. During the photovoltaic process, the HTL acts as a passivation layer between the crystal surfaces of the perovskite and the anode, playing a crucial role in the stability of the solar cell. Poor interfacial quality with halide perovskites can cause easy degradation of solar cells when exposed to sunlight, heat, and moisture.^10,11^ Moreover, a type II band alignment at the HTL/halide perovskite interface is required to allow efficient hole extraction and block electron transport from the perovskite to the anode. A small valence band offset (VBO) with the valence band minimum (VBM) of the halide perovskite slightly above that of the HTL, along with a large conduction band offset (CBO), contributes to the cell’s optimal PCE.^12^ Additionally, the high hole mobility of HTL materials ensures rapid hole transport to the anode, while their good optical transparency minimizes photon loss.^9^

Extensive efforts have been made to select suitable HTLs and optimize the interfacial quality between perovskites and HTLs. NiO and Co_3_O_4_ have been widely recognized as two good HTL materials in PSCs, due to their low-cost synthesis, wide band gaps, high transparency, and favorable type II band alignment with perovskites.^9,13,14^ Additionally, compared to organic HTL materials like poly(triaryl-amine) (PTAA), inorganic metal oxides like NiO and Co_3_O_4_ offer improved stability.^9^ However, degradation issues have still been observed in NiO-based PSCs, which arise from the formation of Ni vacancies at the NiO/perovskite interfaces. The presence of such Ni vacancies can lead to the oxidation of I^–^ ions and deprotonation of MAPbI_3_ at the interface, resulting in the decomposition of MAPbI_3_ on NiO_*x*_ under ambient conditions.^10^ PSCs based on MAPbI_3_ with CoO_*x*_ as the HTL have been reported to exhibit higher PCE compared to those using NiO_*x*_ as the HTL, under similar solar cell structures and operating conditions.^14^ Moreover, they show relatively better stability, maintaining approximately 12% PCE over 1000 h. However, this duration remains insufficient for long-term applications.

Another reason for the instability of PSCs is the intrinsic instability of the halide perovskite cubic structure itself. Presently, PSCs with high PCE have predominantly relied on MAPbI_3_ or FAPbI_3_ inorganic–organic hybrid halide perovskites, due to their suitable band gaps of ∼1.55 and 1.47 eV, respectively, for high light absorption. However, the thermal stability of cubic MAPbI_3_ is low, as the MA^+^ molecular ion size is insufficient to stabilize its perovskite structure, giving a tolerance factor of 0.95.^15^ At temperatures above 85 °C, the MA^+^ cation is released from the lattice, causing a degradation of MAPbI_3_.^16^ This degradation process is further accelerated in humid and illuminated conditions. FAPbI_3_ exhibits a higher tolerance factor of 1.03^15^ due to the larger size of FA^+^. However, the metastable cubic structure is susceptible to changing to a lower-dimensional δ phase at room temperature, especially in moist environments.^17^ To enhance the thermal stability of MAPbI_3_ and the cubic stability of FAPbI_3_, researchers have explored mixing FA^+^ with MA^+^ at the A site of the halide perovskite. Solar cells based on (MA,FA)PbI_3_ have shown promising results, reaching a PCE of 23.2%.^18^

Here, we conduct a comprehensive theoretical analysis of the band alignments and stabilities of Co_3_O_4_/FA_0.4_MA_0.6_PbI_3_ and NiO/FA_0.4_MA_0.6_PbI_3_ interfaces to be compared with the experimental results of Cui et al.^19^ We compare the performance of Co_3_O_4_ and NiO as HTLs and investigate the impact of the perovskite’s two terminations, FA,MA-I, and Pb-I, on the interfacial properties. We suggest that Co_3_O_4_ is a better HTL material than NiO due to its type II band alignment with small VBOs, higher interfacial adhesion energies, and higher formation energies of interfacial metal vacancies with FA_0.4_MA_0.6_PbI_3_. We also show that FA_0.4_MA_0.6_PbI_3_ shows higher adhesion energies with either Co_3_O_4_ or NiO than MAPbI_3_. This analysis offers insights for the design of stable PSCs by proposing the combination of (MA,FA)PbI_3_ as the absorbing layer and Co_3_O_4_ as the HTL, which holds promise for achieving long-term stability in PSCs.

## 2 Calculation Methods

The VASP code^20^ was used to carry out geometry optimization, energy minimization, and electronic property calculations for bulk, surfaces, and interfaces using density functional theory (DFT). The Perdew–Burke–Ernzerhof (PBE) form of the generalized gradient approximation (GGA-PBE) was employed as the exchange-correlation functional. Atoms were represented by projected augmented wave (PAW) pseudopotentials. A *k*-mesh of 7 × 7 × 7 was used for NiO bulk and 5 × 5 × 5 for bulk Co_3_O_4_. A 9 × 9 × 9 *k*-mesh was used for the bulk Ni and Co. A 5 × 5 × 1 *k*-mesh was used for bulk FA_0.4_MA_0.6_PbI_3_. A 1 × 3 × 1 *k*-mesh was applied for the metal oxide (Co_3_O_4_ or NiO)/FA_0.4_MA_0.6_PbI_3_ interfaces. A plane-wave cutoff energy of 520 eV was used. The force tolerance for structural relaxation was 0.05 eV/Å, and the total energy tolerance was set to 10^–7^ eV.

GGA tends to underestimate band gaps, especially for strongly correlated materials like NiO and Co_3_O_4_. Thus, the HSE functional with spin polarization was applied to find the electronic properties. In previous research, the band gaps of bulk NiO and Co_3_O_4_ were found to be 4.3 and 1.6 eV,^21−25^ respectively, using Heyd–Scuseria–Ernzerhof (HSE) and sX hybrid functionals,^26,27^ rather than by GGA + U,^28^ and these values are close to their experimental values.^29^ In this report, the electronic structure, band alignments, adhesion energies, and defect formation energies of the interfaces between perovskites and NiO or Co_3_O_4_ were calculated using the HSE functional, with supercells containing approximately 250 atoms. A Hartree–Fock exchange fraction of α = 0.2 is used, which gives band gaps of 1.46, 1.6, and 3.2 eV for FA_0_._4_MA_0_._6_PbI_3_, Co_3_O_4_, and NiO, respectively, with some compromise on the band gap of NiO. The calculated band gaps, VBMs, and CBMs by HSE functional are shown in Table 1.

**Table 1:** Valence Band Maximum (VBM), Conduction Band Minimum (CBM) Energies w.r.t Vacuum Level, Band Gap, Refractive Index (*N*), Optical Dielectric Constant (ε_∞_), and Interfacial Pinning Factor *S*(25)

	VBM (eV)	CBM (eV)	Band gap (eV)	*n*^2^ = ε_∞_	*S*
NiO	5.84	2.64	3.2	4.75	0.73
Co_3_O_4_	5.29	3.69	1.6	5.06	0.70
FA_0.4_MA_0.6_PbI_3_	5.57	4.11	1.46	4.66	0.75

The band edge energies of the bulk perovskite lie within the bounds of the experimental results when sample stoichiometry is taken into account.^1^ On the other hand, they disagree with the HSE results found by Tao et al.^30^ These latter authors give an interesting analysis of the band extrema energies of bulk perovskites from the perspectives of photoemission, sample stoichiometry,^1^ and theoretical calculation, bearing in mind the conflicts in existing literature. We have included van der Waals corrections in our results for the organic fragments.^31^

## 3 Results

### 3.1 Band Alignment Analysis

Electronic transport is primarily driven by the photoexcited electrons and holes in the conduction and valence band edges, respectively. Therefore, measuring the energies of these band edges with respect to the vacuum level is crucial. These were determined by ultraviolet photoemission spectroscopy (UPS) recently by Cui et al.^19^ The relative experimental energies of band edges for these buried interfaces were also determined by X-ray photoemission spectroscopy (XPS) and approximated by Kraut’s core-level method.^32^ The Kraut method, also called the core-level method, assumes that the energy difference between the VBM and the core level at the interface remains consistent with that in the bulk, providing the band alignment between two bulk materials rather than the alignment after interface formation. The interface creates a shared environment that places both materials within the same system, allowing for a direct comparison of their core-level energy positions. By combining this with the energy difference between the VBM and the core level in each bulk, the band alignment between the two bulk materials can be determined. This method was extended approximately to systems of varying flexibility and stiffness around the interface. Although EA and IP can approximately estimate the VBM and CBM positions relative to the vacuum level, thus determining the VBO and CBO, the presence of possible surface states within the band gap may influence the VBM and CBM positions. In this sense, the core-level method is a more reliable approach for achieving band alignment.

**Figure 1:**
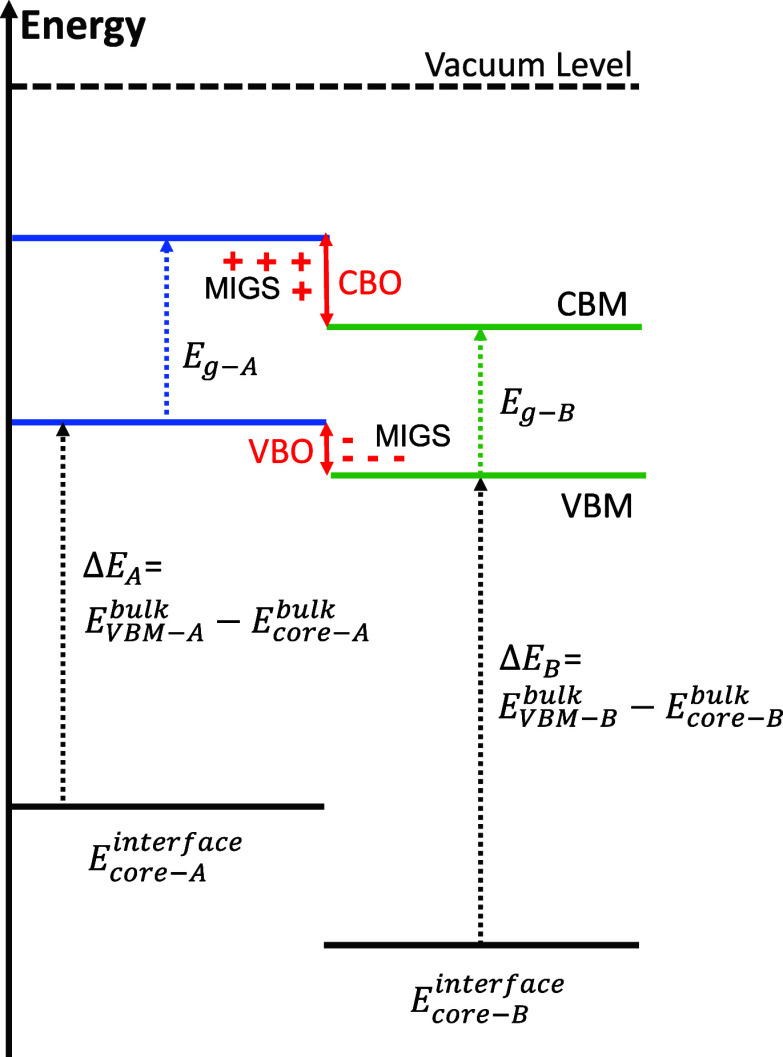
Schematic diagram of calculated interface band alignment using the core-level method. Positive (“+”) and negative signs (“–”) refer to electron deficit and excess of induced gap states, respectively. The metal induced gap states (MIGS) are shown for the type II interface case.

A schematic diagram of calculating interface band alignment is shown in Figure 1. The two materials forming the interface are represented by A and B, where A represents the HTLs, NiO or Co_3_O_4_, and B refers to halide perovskite.  and  are the energies of the VBM in bulk A and B, respectively.  and  represent the energies of the core level in bulk A and B. Δ*E*_A_ and Δ*E*_B_ are the energy differences between the core level and the VBM in bulk A and B.  and  denote the energies of the core level of A and B at the interface. Thus, the VBMs of A and B at the interface are  and , respectively. The CBMs of A and B at the interface are the energy of the VBM at the interface plus their own band gap *E*_g-A_ and *E*_g-B_ in bulk. The energy difference between the VBMs of A and B is the VBO, while the energy difference between the CBMs is the CBO. In this study, Pb-1s, Ni-1s, and octahedral Co-1s orbitals were selected as the core level orbitals in FA_0.4_MA_0.6_PbI_3_, NiO, and Co_3_O_4_, respectively. The type II band alignment, where the VBM of A is slightly above B, facilitates hole extraction from B to A. The higher CBM of A compared to that of B helps to block electron transport from B to A.

The band alignments of ideal semiconductor–semiconductor interfaces are found using the metal induced gap states (MIGS) model.^33,34^ Here, an interface dipole creates charge transfer across the interface. MIGS are the induced gap states formed at metal–semiconductor interfaces by states in the metal tunneling into the semiconductor band gap. Likewise, MIGS arise at semiconductor–semiconductor interfaces by the states of one semiconductor falling energetically within the band gap of the other semiconductor (Figure 1), tunneling a few angstroms into the latter. From the charge neutrality level (CNL) model, the CBO of the semiconductor–semiconductor interface can be written as^35,36^
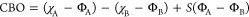
1where χ_A_ and χ_B_ are the electron affinities of semiconductors A and B, respectively, and Φ_A_ and Φ_B_ are their CNLs, relative to the vacuum level. *S* represents the pinning factor.^35^*S* = 1 corresponds to an interface fully pinned by the MIGS, whereas *S* = 0 corresponds to an unpinned interface.^35,36^ The VBO is found from the difference in ionization potential.

*S* varies with the band gap of the phase with smaller gap, the perovskite in this case, where^35^
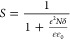
2

This is approximated by the semiempirical equation,^37,38^
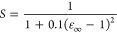
3which gives *S* ∼ 0.67–0.71 for the oxides (Table 1). Co_3_O_4_ has a similar refractive index and ε_∞_ in the optical gap to that of NiO, despite its narrower gap. *S* for the perovskite is ∼0.75, largely because of its relatively small ε_∞_ because of the openness of its lattice.

Halide perovskites have a high tolerance for defects due to their soft lattice structure and as their defect states typically lie near their band edges.^5,39^ This causes the interfacial band offset to be controlled mainly by the intrinsic MIGS. The shift in band edge energies before and after the contact between the HTL and halide perovskite depends primarily on the intrinsic interfacial dipole. The effect of defect states from the perovskite on the band offset is small, whereas that from the oxide could still be significant.

When the valence band is entirely filled and the conduction band is empty, charge neutrality is achieved. Consequently, filling a gap state leads to a local excess of net charge, whereas leaving a gap state empty gives a deficit of local net charge. Gap states near the bottom of the gap can be easily filled with a small electron excess (“–” in Figure 1), whereas those higher in the gap easily remain empty with a small electron deficit (“+” in Figure 1). The band discontinuity induces gap states and associated charges on both sides of the interface.^31−33^ The resulting interfacial dipole causes the band edges to shift, aiming to cancel the charges induced by gap states and achieve zero interfacial dipole.

**Figure 2:**
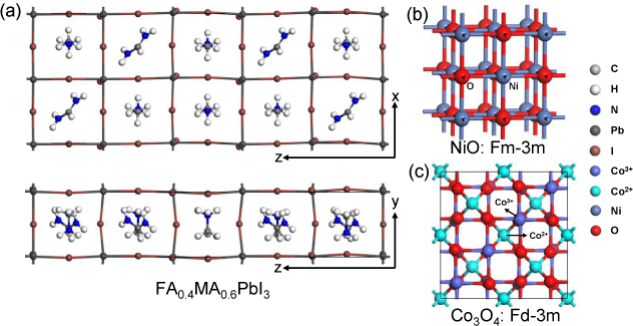
Optimized structures of (a) FA_0.4_MA_0.6_PbI_3_ supercell viewed from *y* and *x* directions, (b) bulk NiO, and (c) bulk Co_3_O_4_.

Figure 2 shows the optimized structures of the supercell FA_0.4_MA_0.6_PbI_3_, bulk NiO, and bulk Co_3_O_4_. The optimized lattice parameters for the supercell FA_0.4_MA_0.6_PbI_3_ are *a* = 11.88 Å, *b* = 5.93 Å, and *c* = 32 Å, for Co_3_O_4_ are *a* = *b* = *c* = 8.07 Å, and for NiO are 4.17 Å. The experimental lattice parameters for Co_3_O_4_ are *a* = *b* = *c* = 8.13 Å, and for NiO are 4.18 Å.^40,41^ The supercell of FA_0.4_MA_0.6_PbI_3_ has a cubic-like structure. Cubic NiO and Co_3_O_4_ have symmetries *Fm*3̅*m* and *Fd*3̅*m*, respectively, and both show antiferromagnetic behavior. Each Ni atom carries a magnetic moment of 1.98 μ_B_. In Co_3_O_4_, tetrahedral Co atoms are in the Co^2+^ state and are coordinated by four oxygen atoms (CoO_4_). Each Co^2+^ ion exhibits a magnetic moment of 2.78 μ_B_, consistent with previous research.^42^ In contrast, the octahedral Co atoms are in the Co^3+^ state and are coordinated by six oxygen atoms (CoO_6_). Co^3+^ ions do not show magnetic moments.

In this study, we constructed Co_3_O_4_/FA_0.4_MA_0.6_PbI_3_ and NiO/FA_0.4_MA_0.6_PbI_3_ interfaces. For the metal oxide side, the Co_3_O_4_ slab is exposed to the (100) surface due to its lower surface energy compared to the (110), (112), and (111) surfaces.^43^ Similarly, the NiO slab is bonded to the nonpolar (100) surface, as it has been reported to be more stable than other frequently studied surfaces, such as (110) and (111).^44^ The perovskite (001) surface is exposed, as it is reported to be a good intermediate for hole transfer to the oxide layer.^45^ Interfaces built with two types of terminations for the perovskite slab, Pb-I and FA,MA-I terminations, are studied. At the interface, the nearest perovskite layer in contact with the metal oxide side is embedded with both MA^+^ and FA^+^.

The average lattice parameters along the *a* and *b* directions of the component slabs are applied to the interfaces. The lattice mismatches for the Co_3_O_4_/FA_0.4_MA_0.6_PbI_3_ and NiO/FA_0.4_MA_0.6_PbI_3_ interfaces are 5% and 3%, respectively. These mismatches are relatively large. However, achieving smaller lattice mismatches would require the application of a much larger supercell, significantly increasing the computational cost and time for calculations using the HSE functional. Moreover, the applied surfaces have shown good stability in experiments.^43−45^ In experiments, sometimes a buffer layer is applied in-between NiO and MAPbI_3_ to lower the lattice mismatch. Despite the large lattice mismatches, perovskites have a soft lattice structure, which allows the bonding to be maintained even under large strain. However, to prevent too large strain on one side, which could influence the interfacial properties and stability, the average lattice parameters are applied for interfaces in both cases. Each interface model comprises two interfaces that have identical characteristics and atomic configurations, resulting in periodicity in the *x*, *y*, and *z* directions. No vacuum slab is involved. The thickness of each component slab is selected to maintain the central layers as bulk-like structures. These bulk layers are situated away from the interface, giving minimal influence on the interfacial region. By preserving bulk-like properties within the bulk layers, the configuration and electronic properties of the two interfaces in the model remain unaffected by each other.

**Figure 3:**
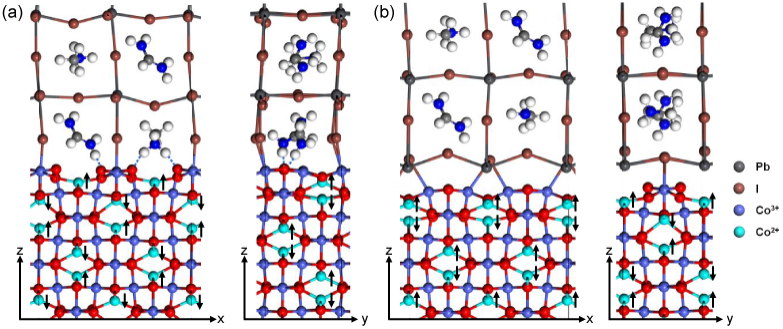
Optimized structures of Co_3_O_4_(100)/FA_0.4_MA_0.6_PbI_3_(001) interface with (a) FA,MA-I termination and (b) Pb-I termination for FA_0.4_MA_0.6_PbI_3_, viewed from *y* and *x* directions, respectively. Black arrows mark directions of the spin polarization. Blue dot lines represent hydrogen bonds.

Figure 3 shows the optimized structure of the Co_3_O_4_(100)/FA_0.4_MA_0.6_PbI_3_(001) interface. In Figure 3a, the interface has FA,MA-I termination for FA_0.4_MA_0.6_PbI_3_. The interface has dimensions of *a* = 11.75 Å, *b* = 5.86 Å, and *c* = 44 Å, with a total of 238 atoms. The interfacial interactions primarily arise from FA/MA–O interactions and I–Co interactions. Moreover, MA^+^ and FA^+^ are attracted to oxygen ions and undergo rotations, allowing the orientation of hydrogen toward oxygen to form H–O hydrogen bonds. The hydrogen and van der Waals bonds from the large organic molecular ions also contribute to the interfacial stability. The interface in Figure 3b has Pb-I termination for FA_0.4_MA_0.6_PbI_3_. This interface has dimensions of *a* = 11.75 Å, *b* = 5.86 Å, and *c* = 51.6 Å, with a total of 264 atoms. Interfacial Pb ions are drawn closer to the interface due to their interactions with oxygen atoms. Bonding between interfacial I and Co atoms further enhances the interfacial interaction. Various interfacial bonding configurations were tested, including positioning the perovskite slab with Pb above O or Co. However, regardless of the initial configuration, after relaxation, the Pb atom consistently shifted to the position seen in Figure 3b. This suggests that this configuration is the most stable interfacial position.

**Figure 4:**
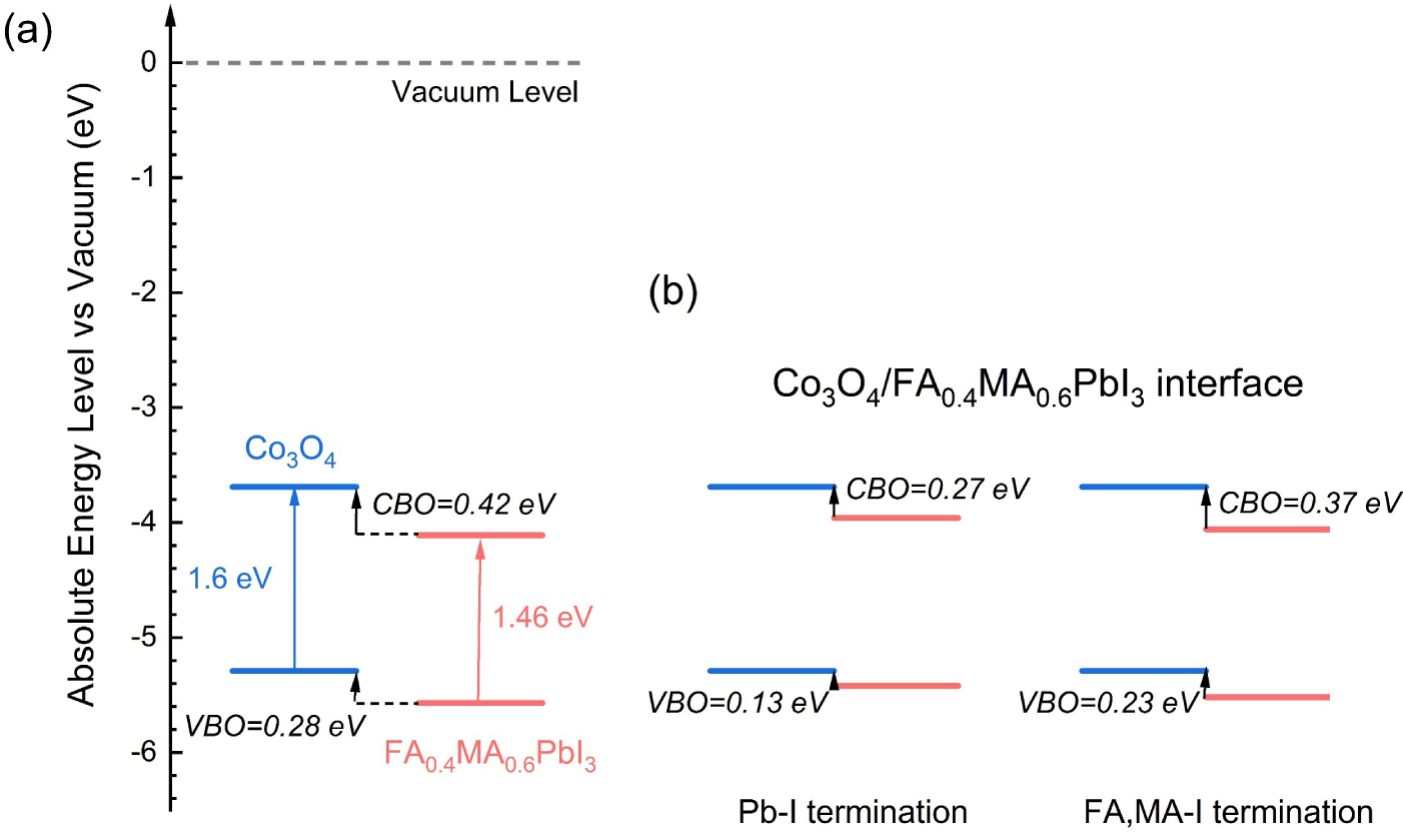
Calculated band alignments of Co_3_O_4_ and FA_0.4_MA_0.6_PbI_3_ (a) using EA and IP and (b) the core-level method. Relevant experimental research is cited.^19^

Figure 4 shows the band alignments of Co_3_O_4_ and FA_0.4_MA_0.6_PbI_3_ using EA/IP and the core-level method. Co_3_O_4_ has IP and EA values of 5.29 and 3.69 eV, respectively. This EA value closely aligns to values reported in previous experimental studies, about 3.65 eV.^46^ FA_0.4_MA_0.6_PbI_3_ has IP and EA values of 5.57 and 4.11 eV, respectively. Through EA and IP, the band alignment is type II with a VBO of 0.28 eV and a CBO of 0.42 eV. Bader charge calculation shows that there is an electron transfer around 0.53 from Co_3_O_4_ to FA_0.4_MA_0.6_PbI_3_. Using the core-level method, the VBO and CBO are 0.13 and 0.27 eV for the Pb-I termination and 0.23 and 0.37 eV for the FA,MA-I termination. A previous study^14^ showed that when the VBO exceeds 0.1 eV, the PCE begins to decrease. This is primarily due to the reduction in open-circuit voltage (*V*_oc_). As the VBO increases, the band gap at the interface decreases, resulting in a lower *V*_oc_.^14^ Thus, Co_3_O_4_/FA_0.4_MA_0.6_PbI_3_ with Pb-I termination for the perovskite is better suited to high-performance PSC fabrication than those with FA,MA-I perovskite termination.

**Figure 5:**
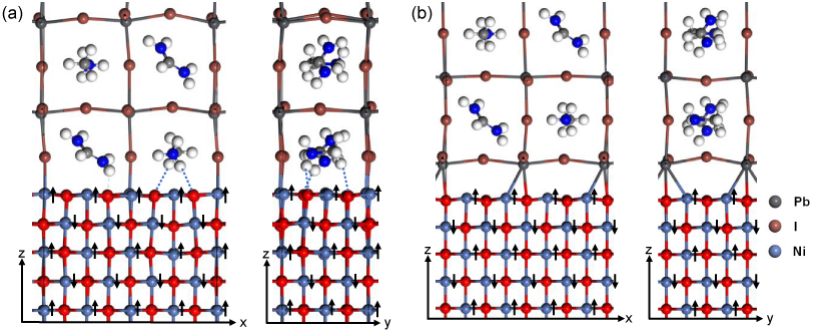
Optimized structures of NiO(100)/FA_0.4_MA_0.6_PbI_3_(001) interface with (a) FA,MA-I termination and (b) Pb-I termination for FA_0.4_MA_0.6_PbI_3_, viewed from *y* and *x* directions, respectively. Black arrows mark directions of the spin polarization. Blue dot lines represent hydrogen bonds.

To compare NiO and Co_3_O_4_ as HTL materials, we further constructed NiO(100)/FA_0.4_MA_0.6_PbI_3_(001) interfaces, as shown in Figure 5. For interfaces with FA,MA-I termination as seen in Figure 5a, the interfacial stability mainly arises from I–Ni bonds. The interface has dimensions of *a* = 11.84 Å, *b* = 5.89 Å, and *c* = 50.34 Å, with a total of 290 atoms. As in Co_3_O_4_/FA_0.4_MA_0.6_PbI_3_, the interfacial FA^+^ and MA^+^ ions also undergo rotations to allow the formation of hydrogen bonds between the H and O atoms. This process contributes to stability, along with van der Waals interactions from the presence of large organic molecular ions. For the interface with Pb-I termination shown in Figure 5b, the interfacial Pb atom, attracted by O, shifts toward the interface. The interface has dimensions of *a* = 11.84 Å, *b* = 5.89 Å, and *c* = 56.53 Å, with a total of 302 atoms. Interfacial stability is mostly contributed by interfacial Pb–O covalent bonds. Interfacial Pb–Ni metallic bonds are also observed.

**Figure 6:**
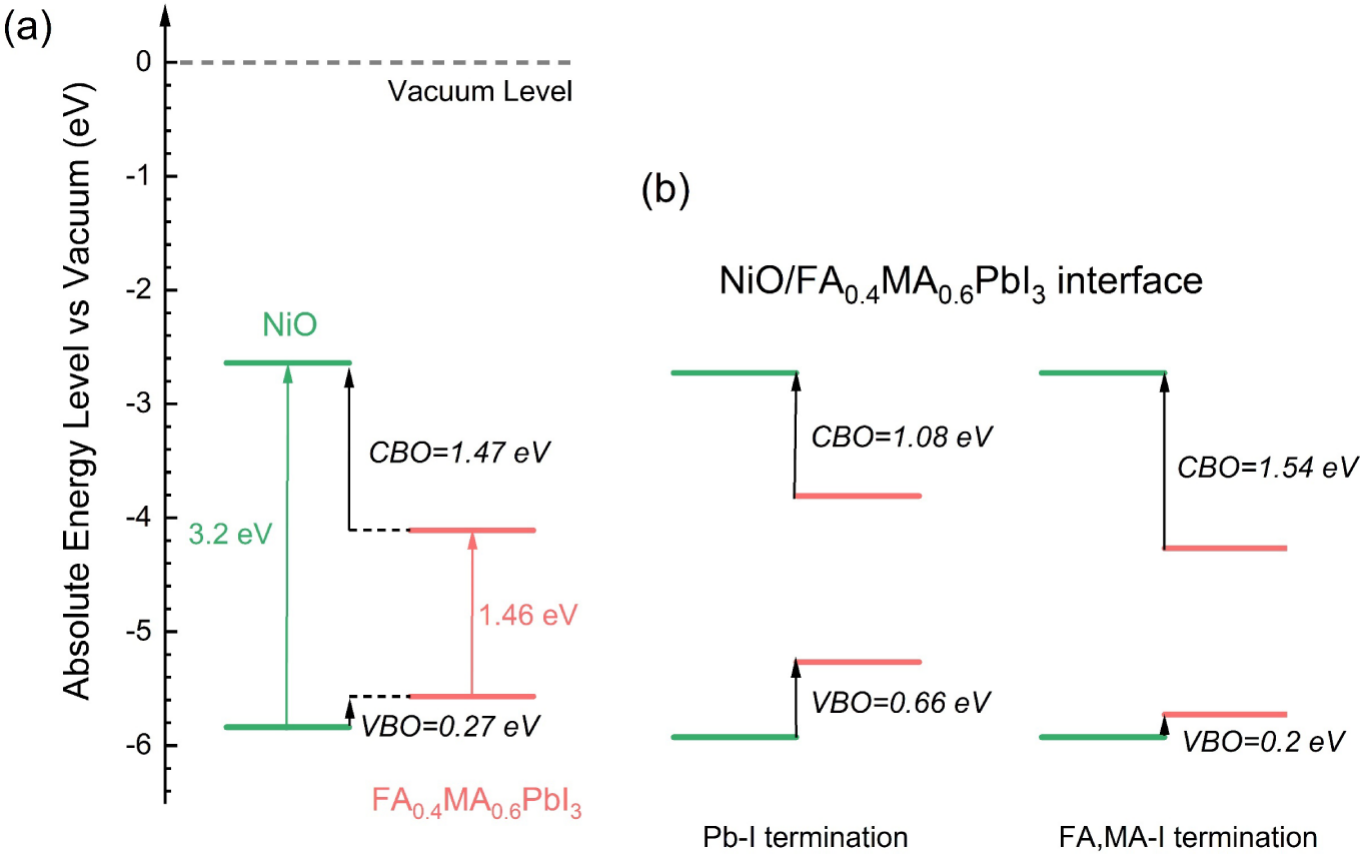
Calculated band alignments of NiO and FA_0.4_MA_0.6_PbI_3_ (a) using EA and IP and (b) the core-level method. Relevant experimental research is cited.^19^

Figure 6 shows the band alignment of NiO and FA_0.4_MA_0.6_PbI_3_. NiO has IP and EA values of 5.84 and 2.64 eV, respectively. The band alignment exhibits a type I configuration with a VBO of 0.27 eV and a CBO of 1.47 eV. Bader charge calculations reveal a transfer of 0.06 electrons from FA_0.4_MA_0.6_PbI_3_ to NiO at the NiO/FA_0.4_MA_0.6_PbI_3_ interface with Pb-I termination for the perovskite. The core-level method gives a type I band alignment with a VBO of 0.66 eV and a CBO of 1.08 eV. Similarly, at the NiO/FA_0.4_MA_0.6_PbI_3_ interface with FA,MA-I termination for the perovskite, there is a transfer of 0.21 electrons from FA_0.4_MA_0.6_PbI_3_ to NiO. The core-level method also gives a type I band alignment, but with a smaller VBO of 0.2 eV and a larger CBO of 1.54 eV. The direction of charge transfer at the interface is determined by the relative positions of the Fermi levels of the component surfaces before contact. Due to the presence of surface states, the Fermi level is not positioned at the center of the band gap, leading to different charge transfer directions in Co_3_O_4_-based and NiO-based interfaces. These type I band alignments are not efficient for hole extraction. Experimental studies on NiO/MAPbI_3_ interfaces have shown type II band alignment, with a VBO ranging from 0.1 to 0.4 eV. However, this research and previous studies have indicated that in the absence of interfacial Ni vacancies or dopants (such as Mg or Li), the NiO/MAPbI_3_ interface tends to shift toward type I band alignment, which hinders hole extraction.^47^ Similar conclusions have been drawn for the interface between NiO and the orthorhombic phase of MAPbI_3_.^48^ The experimentally observed type II band alignment^10^ may be influenced by interfacial Ni defects. Although these defects contribute to interface degradation, they may also play a crucial role in achieving type II band alignment. This trade-off makes NiO a less ideal HTL.

### 3.2 Interfacial Stability

The stability of an interface has a great impact on its formation. One measure to assess interfacial stability is the adhesion energy, which quantifies the energy gained from the reformation of interfacial bonds when two slabs are joined to create an interface. The adhesion energy of metal oxide/perovskite interfaces in this study can be expressed as
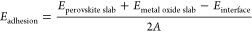
4where *E*_interface_ refers to the total energy of the optimized interface. *E*_perovskite slab_ and *E*_metal oxide slab_ represent the total energies of the perovskite slab and metal oxide slab, respectively, which are calculated from fully relaxed perovskite and metal oxide slabs. *A* is the interfacial area. The lattice parameters of the metal oxide and perovskite slabs remain the same as those at the interface. For instance, in the case of the Co_3_O_4_/FA_0.4_MA_0.6_PbI_3_ interface, the adhesion energy is determined by subtracting the total energy of the optimized interface from the sum of the total energies of the individual optimized Co_3_O_4_(100) and FA_0.4_MA_0.6_PbI_3_(001) slabs. The total energy of the interface is lower than the sum of the energies of the individual slabs due to the adhesion energy. Thus, adhesion energies are presented as positive values here, with a higher adhesion energy implying a stronger binding between the metal oxide and perovskite components at the interface and a higher energy required to break the interfacial bonds, leading to higher interfacial stability.

**Figure 7:**
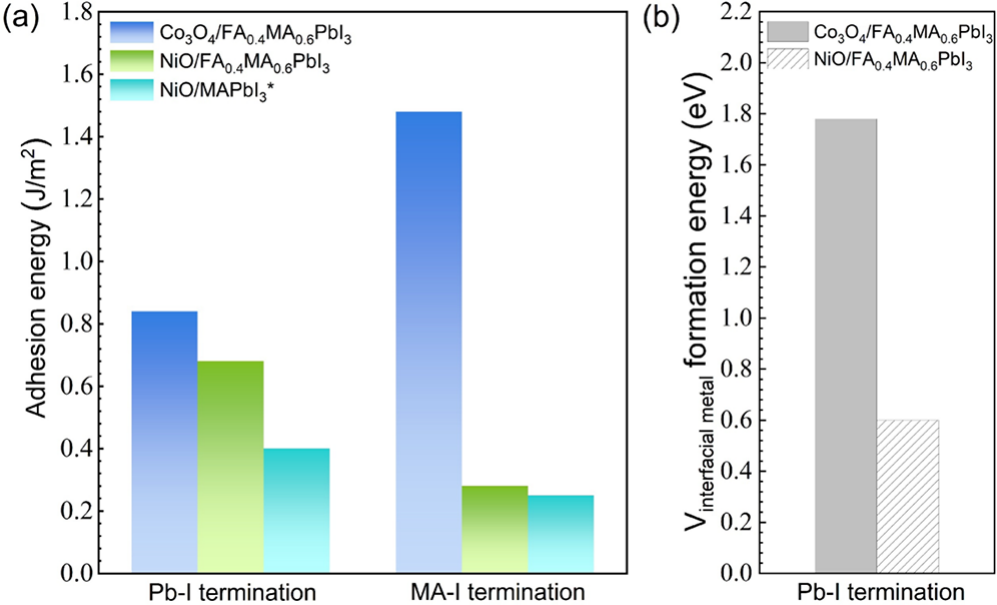
(a) Comparison of adhesion energies of Co_3_O_4_/FA_0.4_MA_0.6_PbI_3_, NiO/FA_0.4_MA_0.6_PbI_3_, and NiO/MAPbI_3_ interfaces with Pb-I or FA,MA-I termination for the perovskite. *Adhesion energies of NiO/MAPbI_3_ are from Li et al.^47^ (b) Formation energies per interfacial Ni vacancy in NiO/FA_0.4_MA_0.6_PbI_3_ or per interfacial octahedral Co vacancy in Co_3_O_4_/FA_0.4_MA_0.6_PbI_3_.

Figure 7a presents a comparison of the adhesion energies of Co_3_O_4_/FA_0.4_MA_0.6_PbI_3_ and NiO/FA_0.4_MA_0.6_PbI_3_ interfaces, considering both Pb-I and FA,MA-I terminations for the perovskite. Adhesion energies of NiO/MAPbI_3_ are taken from Li et al.^47^ Co_3_O_4_/FA_0.4_MA_0.6_PbI_3_ with FA,MA-I termination for the perovskite exhibits an adhesion energy of 1.48 J/m^2^, 0.64 J/m^2^ more than those with Pb-I termination. NiO/FA_0.4_MA_0.6_PbI_3_ interfaces with Pb-I termination for the perovskite display an adhesion energy of 0.68 J/m^2^, 0.4 J/m^2^ higher than those with FA,MA-I termination. These findings suggest that Co_3_O_4_ interfaces with FA,MA-I termination in the perovskite exhibit higher interfacial stability compared to those with Pb-I termination. Conversely, NiO interfaces show greater stability when the perovskite has Pb-I termination rather than FA,MA-I termination. NiO/FA_0.4_MA_0.6_PbI_3_ interfaces show higher adhesion energies than NiO/MAPbI_3_ interfaces (0.68 eV vs 0.4 eV), indicating the advantage of using FA_0.4_MA_0.6_PbI_3_ as the light absorber. Likely due to the large lattice mismatch between NiO or Co_3_O_4_ and FA_0.4_MA_0.6_PbI_3_, the overall adhesion energies are not high. However, utilizing Co_3_O_4_ as the HTL and FA_0.4_MA_0.6_PbI_3_ as the light-absorbing layer results in both smaller VBOs and higher adhesion energies compared to NiO-based interfaces. This makes Co_3_O_4_ a more promising candidate for fabricating PSCs with improved long-term stability.

To compare the ease of formation of the interfacial metal vacancy, we further calculated the formation energies per interfacial Ni vacancy in NiO/FA_0.4_MA_0.6_PbI_3_ and per interfacial octahedral Co vacancy in Co_3_O_4_/FA_0.4_MA_0.6_PbI_3_ using the formula:
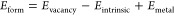
5

*E*_vacancy_ represents the total energy of the interface with a single interfacial Ni or Co vacancy, *E*_intrinsic_ refers to the total energy of the intrinsic interface, and *E*_metal_ is the energy of a Ni or Co atom in their *Fm*3̅*m* bulks. One interfacial Ni atom is introduced in NiO/FA_0.4_MA_0.6_PbI_3_ with a Pb–I-terminated perovskite. Similarly, one interfacial octahedral Co atom is introduced in Co_3_O_4_/FA_0.4_MA_0.6_PbI_3_. The energy is then calculated by the HSE functional for the relaxed interface with a single interfacial Ni or Co vacancy.^48^

Figure 7b shows formation energies per interfacial Ni vacancy in NiO/FA_0.4_MA_0.6_PbI_3_ or per interfacial octahedral Co vacancy in Co_3_O_4_/FA_0.4_MA_0.6_PbI_3_. At the NiO/FA_0.4_MA_0.6_PbI_3_ interface, the formation energy per interfacial Ni vacancy is 0.6 eV. This low formation energy illustrates that Ni vacancies are easy to form during the fabrication process. However, previous research has indicated that Ni vacancies mainly trigger the redox reaction and deprotonation of the organic molecular ion at the interface under ambient conditions.^10^ This leads to the instability of interfaces and subsequently degrades the performance of solar cells. At the Co_3_O_4_/FA_0.4_MA_0.6_PbI_3_ interface, each interfacial octahedral Co vacancy shows a much higher formation energy of 1.8 eV. This indicates a greater difficulty in forming Co vacancies, thereby impeding the redox reaction and deprotonation process. Environmental conditions, such as heat or light, provide energies for defect formation. This higher vacancy formation energy contributes to the higher interfacial stability under environmental conditions, further suggesting Co_3_O_4_‘s suitability over NiO as an HTL material.

## 4 Conclusions

In this theoretical study, our analysis of the Co_3_O_4_/FA_0.4_MA_0.6_PbI_3_ and NiO/FA_0.4_MA_0.6_PbI_3_ interfaces reveals that Co_3_O_4_ exhibits better characteristics as an HTL compared to NiO, primarily due to three key advantages. First, Co_3_O_4_/FA_0.4_MA_0.6_PbI_3_ interfaces demonstrate type II band alignment with small VBOs of about 0.13 eV. Second, Co_3_O_4_/FA_0.4_MA_0.6_PbI_3_ interfaces exhibit higher adhesion energies than NiO/FA_0.4_MA_0.6_PbI_3_ interfaces (1.48 J/m^2^ vs 0.68 J/m^2^), suggesting better interfacial stability. Third, Co_3_O_4_/FA_0.4_MA_0.6_PbI_3_ demonstrates enhanced stability under environmental conditions due to the higher formation energy of interfacial Co vacancies (1.8 eV) compared to interfacial Ni vacancies in NiO/FA_0.4_MA_0.6_PbI_3_ (0.6 eV). Furthermore, NiO/FA_0.4_MA_0.6_PbI_3_ interfaces show higher adhesion energies than NiO/MAPbI_3_ interfaces. Therefore, we propose that PSCs incorporating FA_0.4_MA_0.6_PbI_3_ as the absorbing layer and Co_3_O_4_ as the HTL could potentially provide efficient hole extraction and long-term stability under environmental conditions.
